# Metagenome-assembled genomes of freshwater *Hyphomicrobium* sp. G-191 and *Methylophilus* sp. enriched from Cedar Swamp, Woods Hole, MA

**DOI:** 10.1128/mra.00608-24

**Published:** 2024-11-14

**Authors:** Yolanda Huang, Rebecca Schomer, María José Rodríguez Reyes, Kyle Costa, Rebecca E. Parales, Rachel Whitaker, Scott C. Dawson

**Affiliations:** 1 Department of Microbiology and Immunology, State University of New York at Buffalo, Buffalo, New York, USA; 2 School of Plant Sciences, University of Arizona, Tucson, Arizona, USA; 3 Molecular Biology Center, University of Central America, Managua, Nicaragua; 4 Department of Plant and Microbial Biology, University of Minnesota, St. Paul, Minnesota, USA; 5 Department of Microbiology and Molecular Genetics, University of California Davis, Davis, California, USA; 6 Department of Microbiology, University of Illinois Urbana-Champaign, Urbana, Illinois, USA; SUNY College of Environmental Science and Forestry, Syracuse, New York, USA

**Keywords:** *Hyphomicrobium*, sediment, *Methylophilus*, C1 metabolism

## Abstract

*Hyphomicrobium* are facultative denitrifying anaerobes capable of using one-carbon compounds as a sole carbon source. *Hyphomicrobium* sp. G-191 was enriched from Cedar Swamp, Woods Hole, Massachusetts, using a selective medium for methanol-utilizing bacteria. We present two draft metagenome-assembled genomes (MAGs) of a *Hyphomicrobium* and a *Methylophilus* species.

## ANNOUNCEMENT

Microorganisms that catabolize C1 compounds are important for global biogeochemistry. There has been increased interest in these organisms in recent years for developing biotechnologies to capture recalcitrant C1 compounds (1). The genus *Hyphomicrobium* is widespread in aquatic, soil-, and plant-associated niches (2, 3). Their unique dimorphic life cycle consists of a sessile prosthecate mother cell and a motile swarmer daughter cell (4). We present a metagenome-assembled genome (MAG) for *Hyphomicrobium* sp. G-191 sequenced during the 2023 Microbial Diversity Course at the Marine Biological Laboratory. An MAG of a *Methylophilus* species was also obtained from the enrichment culture, which is consistent with its ability to utilize methanol as the sole carbon and energy source (5).

Sediments were collected at Cedar Swamp, Woods Hole, MA (GPS coordinates: 41.53,–70.65), and inoculated into a *Hyphomicrobium* selective medium: freshwater base [17.1 mM NaCl, 1.97 mM MgCl_2_*6H_2_O, 0.68 mM CaCl_2_*2H_2_O, 6.71 mM KCl], 20 mM MES [pH 6.15], 1% (vol/vol) SL-10 trace elements (6), 0.2 mM Na_2_SO_4_, 25 µM KPO_4_, 50 mM KNO_3_, and 1% (vol/vol) 13-vitamin solution [riboflavin, biotin, thiamine HCl, ascorbic acid, pantothenate, folic acid, nicotinic acid, 4-aminobenzoic acid, pyridoxine HCl, lipoic acid, NAD, thiamine pyrophosphate, and cyanocobalamin] with 0.25% (vol/vol) methanol as the sole carbon source. Pfennig bottles were filled to remove headspace, incubated at 30°C until anaerobic growth was observed, and transferred three times into fresh media.

gDNA from the final enrichment was extracted using the Qiagen PowerSoil kit, prepared using the Illumina DNA Prep (M) Tagmentation kit, and sequenced on the iSeq100. Paired-end 150-bp reads were filtered using fastp v0.23.4 to obtain 1,531,161 reads for assembly using metaSPAdes v3.14.4 (k-mer lengths of 21, 33, 55, 77, 99, and 127) (7, 8). Contigs were binned using metaBAT2 v2.15 (9). BBMap and SAMtool v1.14 were used to generate the BAM file (10, 11). Assemblies and bins were assessed using QUAST v5.2.0 and CheckM2 v1.0.1, respectively (12, 13). ANI and taxonomic classification were assigned using GTDB-Tk v2.1.1 (14). Bins were annotated using Prokka v1.14.6 (15). Default parameters were used for all software.

Assembly led to a total of 5,159 contigs of 10,413,222 bp and two bins. MAG1 is 2,392,033 bp in total length with 168 contigs, N50 of 19,324 bp, and a GC content of 51.4%. MAG2 has a total length of 3,312,584 bp, 229 contigs, N50 of 24,902 bp, and a GC content of 60.3%. MAG1 has 88.41% completeness with 0.36% contamination and the highest average nucleotide identity (ANI) of 98.91% to a deposited MAG (GCA_008015755.1, Bin_55_2) classified as *Methylophilus* sp.. MAG2 has 90.6% completeness with 1.59% contamination and an ANI of 83.99% to the type strain, *Hyphomicrobium denitrificans* ATCC 51888 (GCF_000143145.1).

We identified 3,204 protein-coding sequences for *Hyphomicrobium* sp. G-191, including predicted genes for methanol and formaldehyde utilization, flagellar motility, chemotaxis, denitrification, and aromatic hydrocarbon degradation. Seven putative methyl-accepting chemotaxis proteins were identified as well as two copies of the chemotaxis signal transducer, CheY.

## Data Availability

This sequencing project has been deposited in the NCBI under the BioProject accession number PRJNA1110591. Raw sequencing reads are available in the Sequence Read Archive (SRA) under the accession number SRR29039226. The MAGs annotated using the NCBI pipeline are available under the accession numbers SAMN41425349 and SAMN41425350. The MAGs annotated using prokka are available on Figshare: https://doi.org/10.6084/m9.figshare.26981488.v1.
